# The obesogenic side of Genistein

**DOI:** 10.3389/fendo.2023.1308341

**Published:** 2023-11-30

**Authors:** Jia Xiang, Ronald Mlambo, Progress Dube, Oleen Machona, Ibrahim Shaw, Yimer Seid, Yongju He, Min Luo, Tingting Hong, Binsheng He, Wenhu Zhou, Songwen Tan

**Affiliations:** ^1^ Academician Workstation, Changsha Medical University, Changsha, China; ^2^ Xiangya School of Pharmaceutical Sciences, Central South University, Changsha, Hunan, China; ^3^ Simon Mazorodze School of Medical and Health Sciences, Great Zimbabwe University, Masvingo, Zimbabwe; ^4^ School of Materials Science and Engineering, Central South University, Changsha, Hunan, China; ^5^ Department of Nephrology, The Second Xiangya Hospital, Central South University, Changsha, Hunan, China; ^6^ School of Pharmacy, Changzhou University, Changzhou, Jiangsu, China

**Keywords:** obesity, endocrine disruptor, adipogenesis, Genistein, peroxisome proliferator-activated receptor gamma

## Abstract

Genistein (GN) has been highly recommended for its medicinal properties like anticancer, antidiabetic, antihyperlipidemic, antiviral, and antioxidant activities among others. Recently, scientists realized that Genistein is an endocrine disruptor. It is an obesogen that interferes with the endocrine system causing obesity through many mechanisms like inducing adipocyte differentiation, lipid accumulation, and transformation of some stem cells into adipocytes (bone marrow mesenchymal stem cells for example) *in vitro*. Animal studies show that GN upregulates genes associated with adipogenesis like CCAAT/enhancer binding protein alpha (Cebpα), CCAAT/enhancer binding protein beta (Cebpβ), and PPARγ. *In silico* studies reveal a strong binding affinity for estrogen receptors. All these findings were contingent on concentration and tissues. It is beyond dispute that obesity is one of the most frustrating medical conditions under the sun. The pathophysiology of this disease was first attributed to a high-calorie diet and lack of physical activity. However, studies proved that these two factors are not enough to account for obesity in both children and adults. This mini review highlights how Genistein interaction with the peroxisome proliferator-activated receptor gamma protein can cause obesity.

## 1 Introduction

Genistein (GN) is a well-known bioflavonoid, an isoflavone to be specific, that is found in many food and plant sources (**Table 1**). Among the sources, soybean is a major source of GN with a concentration ranging from 5.6-276 mg/100g in fully mature soybeans (2, 3). Nowadays, An increasing number of studies indicate that certain natural compounds offer various benefits to the human health (4, 5). GN supplements are on the market particularly in Asia due to their health promoting effects. Evidence is piling up that GN has anti-inflammatory (6), anticancer (7, 8), anti-diabetic (9), anti-hyperlipidemic (10), antiviral (8, 11), antimicrobial (12), antifungal (13), cardioprotective (2, 14), nephroprotective (15), antioxidant (16, 17), and anti-obesity (18) properties among others. Surprisingly, recent studies revealed that GN is a potential endocrine disruptor (ED) that interferes with estrogen receptors (ERs) hence interfering with the endocrinology system (19, 20). Scientists assert that GN, a phytoestrogen, is an obesogen (21) since it induces adipogenesis in a dose-dependent fashion.

**Table 1:** Common food sources of Genistein.

Source	Concentration	References
**Tofu yogurt**	12.3 mg/half cup	(1)
**Tofu soft**	10.1 mg/3 ounces	(1)
**Soy sausage**	6.9 mg/3 links	(1)
**Soy cheese cheddar**	0.6 mg/ounce	(1)
**Unprepared soy burger**	3.5 mg/patty	(1)
**Alcohol washed soy protein concentrate**	5.8 mg/3.5 ounces	(1)
**Aqueous washed soy protein concentrate**	52.8 mg/3.5 ounces	(1)
**Low-fat soy milk**	3.7 mg/cup	(1)
**Dry roasted soybeans**	21.2 mg/ounce	(1)
**Boiled green soybeans**	6.3 mg/half cup	(1)
**Miso**	32 mg/half cup	(1)
**Cooked tempeh**	18 mg/3 ounces	(1)
**Tempeh**	30.7 mg/3 ounces	(1)
**Mature boiled soybean seeds**	26.9 mg/half cup	(1)

(Linus Pauling Institute. Available online: https://lpi.oregonstate.edu/mic/dietary-factors/phytochemicals/soy-isoflavones#source).

Obesity has become a global health crisis (22), and while the common explanation attributes it to a high-calorie diet and lack of physical activity, this explanation falls short in accounting for the increasing rates of obesity in both adults and children over the past few decades (23, 24). This suggests that additional factors must play a significant role. Researchers have found that EDs add to these factors. EDs are xeno-substances that interfere with hormone functions and have become widespread in our environment, exposing humans daily through various means like ingestion, inhalation, and skin contact. Scientific evidence has linked EDs exposure to obesity in laboratory animals (25, 26) and has shown associations with obesity in humans (27, 28). These substances, known as obesogens, cause adipogenesis and obesity through various mechanisms in living organisms. The environmental obesogen theory posits that exposure to obesogens can predispose individuals to obesity, potentially contributing to the obesity epidemic (29). Furthermore, the effects of EDs and obesogen exposure may extend to future generations, a concept referred to as ‘generational toxicology.’ Regulators currently do not consider this aspect in risk assessment, but it could be another significant factor contributing to the obesity crisis and the rise of noncommunicable diseases worldwide (30).

Generally, obesogens have the potential to stimulate the growth of adipocytes and the accumulation of lipids in the body through various mechanisms, including increasing adipocytes number, enlarging adipocytes, disrupting hormonal regulation of fat tissue, affecting appetite control, altering metabolic rate, favoring calorie storage, and impacting insulin sensitivity in various organs. At the cellular level, obesogens can disrupt the endocrine system by interfering with peroxisome proliferator-activated receptors (PPARs) and steroid receptors, which are nuclear transcriptional regulators involved in lipid regulation and fat cell proliferation. This interference can lead to changes in gene expression that ultimately contribute to obesity (31).

Genistein is a recognized substance that interferes with the endocrine system by attaching to estrogen receptors and imitating the effects of naturally occurring sex hormones (32). In fact, GN serves as a binding molecule with high affinity for several nuclear receptors, such as estrogen receptors (ERs), androgen receptor (AR), peroxisome proliferator-activated receptor gamma (PPARγ), liver X receptors (LXRs), pregnane X receptor (PXR), as well as membrane-bound forms of ERs and AR (33). ERs exhibit a high degree of versatility, as they can interact with molecules of diverse molecular structures. The body is exposed to a wide array of such compounds through medical treatments, environmental factors, and nutrition. When it comes to nutrition, the prevalence and abundance of estrogenic substances in our food raise the question of the significance of daily consumption of regulators that influence ER activity on our overall health (34).

This mini review aims to highlight how Genistein interaction with the peroxisome proliferator-activated receptor gamma protein can cause obesity.

## 2 GN as an ED *in vivo*

Genistein influences the regulation of adipose tissue deposition and expansion, processes governed by hormones. However, there is limited knowledge regarding how early-life exposure to GN might impact metabolic balance in adulthood. Scientists conducted a study involving rat pups exposed to GN from postnatal day 1 to 22 to simulate GN levels in infants fed soy formula. The results revealed that female rats exposed to GN exhibited increased fat-to-lean mass ratio, higher fat mass, larger and more numerous adipocytes, and reduced muscle fiber size. Additionally, GN-exposed female rats at postnatal day 22 displayed elevated expression of genes associated with adipocyte formation, such as CCAAT/enhancer binding protein alpha (Cebpα), CCAAT/enhancer binding protein beta (Cebpβ), and PPARγ. Furthermore, Wingless-related MMTV integration site 10b (Wnt10b), a crucial regulator of adipocyte development, exhibited increased methylation and decreased expression in the adipose tissue of GN-exposed female rats. These findings suggest that early-life exposure to GN in rats has gender-specific effects on adiposity, resembling the impact of a high-fat diet after weaning. It emphasizes the significance of considering both timing of exposure and gender when establishing safety guidelines for dietary GN intake during early life (35). Behloul & Wu argue that the GN concentration used in animal and cell experiments is much higher than the human serum GN concentration following daily GN-rich sources intake (36). These researchers rule out the possibility that GN is an endocrine disorder at concentrations lower than 50µM. However, Grossini et al. reported that white adipocyte differentiation is inversely proportional to GN concentration (37). These discrepancies are worthy to clarify through conducting more experiments. The differences could be due to different experimental designs.

In another study, male and female mice that were four weeks old were given daily oral doses of GN ranging from 50 to 200,000 μg/kg per day or 17β-estradiol (E2) at a dose of 5 μg/kg per day for a period of 15 days. Another group of mice was fed a diet containing 800 ppm GN. The results showed that GN increased the size of fat pads in the epididymal and renal regions as well as the size of adipocytes in male mice, but not in female mice, at doses up to 50,000 μg/kg.d or when included at 800 ppm in their diet. This increase in fat was associated with a higher level of insulin resistance in the periphery. The treatments also led to elevated levels of GN in the blood, with concentrations rising from 35 ± 6 to 103 ± 26 nM 12 hours after treatment. Additionally, these treatments resulted in lower levels of triglycerides and cholesterol in the blood. Interestingly, the highest dose of genistein (200,000 μg/kg per day) had a similar effect to 17β-estradiol (E2) in reducing adipose tissue weight. At this dose, GN down-regulated the expression of estrogen receptors, particularly estrogen receptor β (ERβ), and progesterone receptors, while also inducing factors associated with estrogen-dependent adipose tissue development. However, it did not affect the expression of the minimal consensus estrogen-responsive element in ERE-tK-LUC mice, unlike in other tissues such as the lung where it had a positive effect. In contrast, E2 downregulated the expression of most adipogenic factors. Further analysis using gene microarrays revealed that GN had different effects on genes related to fat metabolism and obesity depending on the dose used. The lower dose of genistein induced the expression of the phospholipase A2 group 7 and the phospholipid transfer protein genes, while the highest dose (200,000 μg/kg per day) inhibited them. Importantly, the antiadipogenic action of GN and the down-regulation of adipogenic genes were found to depend on the presence of ERβ. Collectively, these findings indicated that GN at nutritional doses promotes the accumulation of fat in a gender-specific manner, while at pharmacological doses it inhibits fat deposition. These effects involve changes in the expression of estrogen and progesterone receptors and various genes related to fat metabolism and obesity, and they are dependent on the presence of ERβ (38).

### 2.1 *In vitro* studies

Hall and colleagues conducted a cell experiment with an intention to test their hypothesis that the pro- or anti-adipogenic activity of phytoestrogen chemicals is related to the ability to activate PPARγ in adipocytes. These scientists investigated how resveratrol, GN, and daidzein, which are compounds found in soy, impact the process of adipogenesis using 3T3-L1 cells as a model. Simultaneously, they assessed the changes in the expression of PPARγ target genes induced by these compounds through quantitative polymerase chain reaction. Apart from that, they evaluated the agonistic or antagonistic effects of phytoestrogens on PPARγ by measuring their ability to influence the recruitment of transcriptional cofactors to the receptor. Hall and colleagues found that resveratrol significantly exerted it anti-adipogenic effects as it downregulated the genes involved in lipid metabolism, reversed the rosiglitazone-agonistic PPARγ properties, blocked cofactor recruitment to the PPARγ, and antagonized PPARγ-dependent adipocyte differentiation. Daidzein and GN, in contrast, promoted adipogenesis and acted as PPARγ agonists (39).

According to a study conducted by Relic et al., GN potentiated adipogenesis in glucocorticoid-mediated synovial fibroblast cells. In addition, GN transformed synovial fibroblasts into adipocytes. These formed adipocytes did not produce leptin. However, the fat cells were found to produce adiponectin and express perilipin A. Following subjection of the synovial fibroblasts cells to GN in the presence of TNF-*α* for 21-28 days, adipocyte-like cells showed up. These fat lobules stained positively when subjected to oil red. When GN-induced adipocytes were exposed to a PPARγ agonist, rosiglitazone, synergism was observed. Daidzein was found inferior to GN in terms of inducing adipogenesis in synovial fibroblast cells. In short, the inference of this study was that GN-induced adipogenesis involves the inhibition of tyrosine kinase and PPAR-*γ* induction (40). Balbuena-Pecino et al. reported adipocyte differentiation in *Oncorhynchus mykiss* preadipocytes following treatment with different GN concentrations (10 and 100 μM). The preadipocytes treated with 100 μM differentiated more than 10 μM-treated cells forming mature adipocytes. Lipid content in adipocytes treated with 100 μM was highest as compared to the rest of the groups. In other words, lipids accumulated the most in the 100 μM GN-treated adipocytes. All these findings support that GN is an ED that is capable of causing obesity through interacting with the PPARγ protein (41). Similar results were reported by Grossini et al. GN was found to enhance the differentiation of human visceral pre-adipocytes, promoting browning, and leading to a dose-dependent enhancement in cell viability and mitochondrial membrane potential. These effects were also observed in both brown and white adipocytes. However, in white adipocytes, the degree of increase in cell viability was inversely proportional to the dosage (37).

In a different study, scientists aimed to investigate the mechanism behind how GN affects bone marrow-derived mesenchymal stem cells (BMSCs), specifically in terms of inhibiting their differentiation into adipocytes and enhancing osteogenesis. The researchers utilized an MTT assay and found that GN notably boosted the proliferation of BMSCs in a manner dependent on both time and dosage. Furthermore, through reverse transcription-quantitative polymerase chain reaction analysis, it was revealed that GN significantly curtailed the expression of key genes associated with bone formation, including runt-related transcription factor 2 (Runx2), type I collagen (Col I), and osteocalcin (OC). Additional assays showed that a concentration of 20 µm GN hindered the activity of alkaline phosphatase (ALP), an enzyme important in bone formation, while concurrently increasing triglyceride (TG) activity. Lastly, Western blotting demonstrated that pretreatment of BMSCs with 20 µm GN notably elevated the expression of PPARγ protein. This implies that the reduction in PPARγ levels might be a significant factor contributing to the impact of GN on BMSCs, leading to increased cell proliferation, decreased expression of key bone-related genes, diminished ALP activity, and elevated TG activity. Consequently, the findings from this study suggest that GN prompts the differentiation of BMSCs into adipocytes while hampering their potential to become bone cells by upregulating PPARγ expression (42).

### 2.2 *In silico* studies

In silico studies (43, 44) that specifically probe how GN interacts with the PPARγ biomolecule and other hormonal NRs are very limited. Recently, scientists have reported that flavonoids have estrogen activity as EDs *in silico*. In one study that involved GN as one of the four bioflavonoids used in that experiment, The binding affinities of these phytoestrogens for 14 major Eds-NRs varied according to the molecular docking studies. GN exhibited the strongest affinity for both ERα and ERβ receptors, with apigenin coming in as the second most potent binder. However, their binding capabilities were lower in comparison to the affinities of 17β-estradiol. GN and apigenin displayed robust binding to ERβ and moderate binding to ERα. Specifically for ERα, GN showed strong binding, while apigenin exhibited a moderate level of binding (45).

## 3 Mode of action

**Figure 1:**
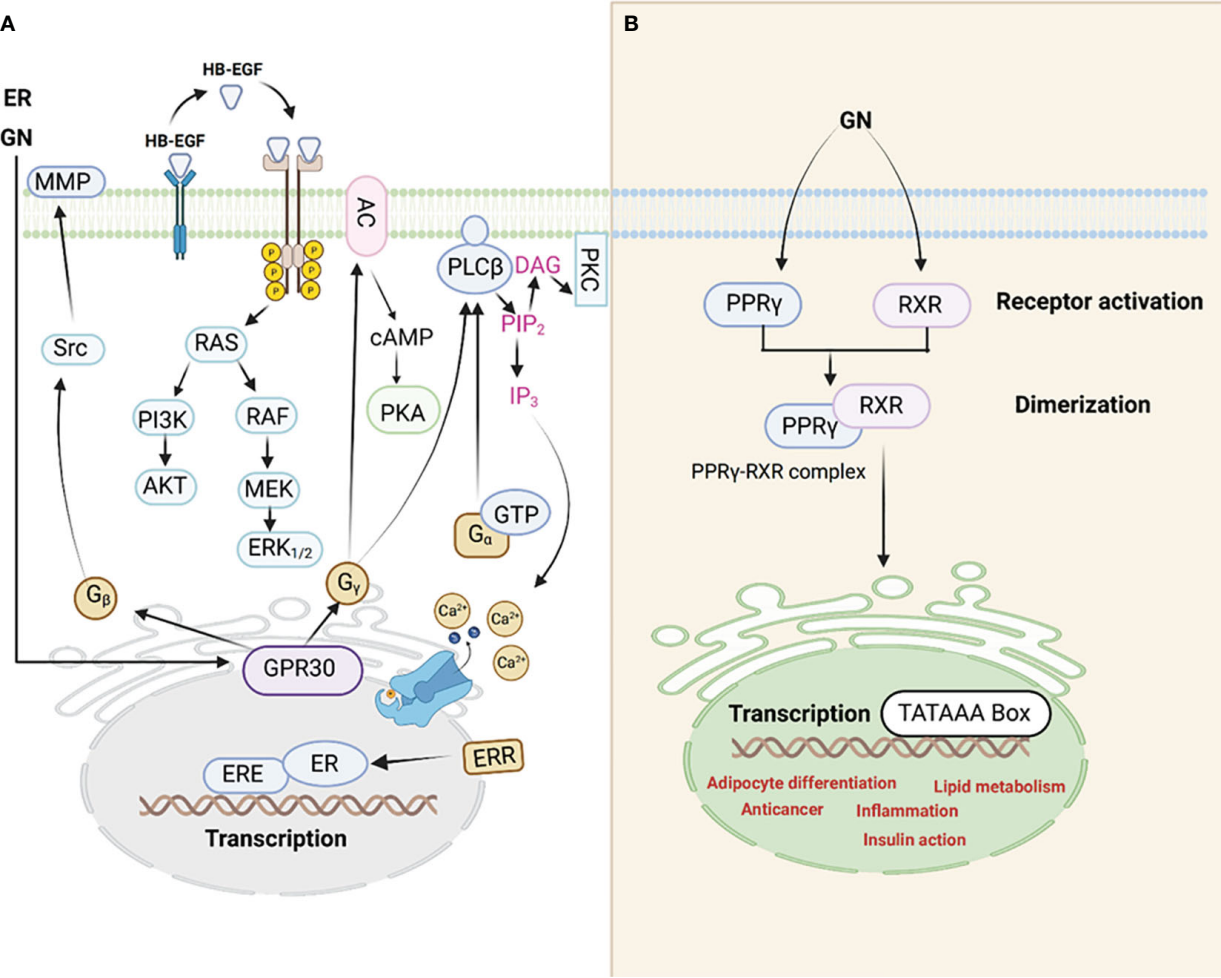
An illustration of the interaction between GN and ER receptors **(A)**. PPARγ signaling pathway **(B)**, GN interacts with the PPARγ leading to adipocyte differentiation among other mechanisms through which GN causes adipogenesis hence obesity.

GN exerts its effects mainly via the PPARγ (**Figure 1**). Scientists assert that this receptor is a pivotal switch for fat metabolism. GN performs its function by controlling estrogen-dependent modulatory processes through a negative interaction with the ERs (46). The phytoestrogen has the capacity to modify cellular activities and curb homeostasis by influencing both the ERs and PPARγ (47). Empirical data obtained through *in vivo* experiments suggest that the modulation of GN through ERs and PPARγ-mediated transcription varies based on factors such as dosage, tissue type, and the receptors’ varying affinities (48, 49). Animal experiments revealed that GN has a dual effect on adipose tissue. At lower doses, it promotes the activation of enzymes closely associated with lipid storage, such as lipoprotein lipase (LPL), possibly through the activation of PPARγ, a regulator of LPL. Conversely, at higher, supra-nutritional, or pharmacological doses, GN significantly inhibits these enzymes, including LPL. This inhibitory mechanism is likely linked to ERs, as its effects closely resemble those induced by estradiol, even though they do not target the same gene promoters (33).

Evidence is piling up which supports the notion that adipogenic signals (GN interaction with the PPARγ, for example) possibly counteract estrogen signaling through PPARγ activation. In addition, scientists reported that estrogens control the action of PPARγ. The interference between ER and PPARγ takes place through various mechanisms. It is noteworthy to understand that when PPARγ is activated, it has the ability to attach itself to the ERE (Estrogen Response Element) found on promoters of genes targeted by estrogen. This binding to DNA is contingent on the specific structure of the ERE within these target gene promoters. As a result of this DNA binding, PPARγ disrupts the functioning of ERs and the expression of genes that are regulated by ER. The PPARγ transcription complex (50), which consists of PPARs and retinoid X receptors (RXRs), can bind to multiple response elements that resemble EREs and contain half-sites with the sequence AGGTCA. However, it does not activate transcription due to the inherent limitations in the promoter structure (51).

### 3.1 PPARγ biochemistry influence in adipose tissue

PPARγ is recognized as the principal regulatory biomolecule of adipogenesis, as it has been demonstrated that introducing PPARγ alone into fibroblasts can effectively initiate the adipogenesis process, and no other elements can trigger adipogenesis unless PPARγ is also present (52). According to animal experiments conducted, it has been observed that PPARγ plays a crucial role in regulating adipogenesis, lipid and glucose metabolism. It is scientifically proven through these experiments that when PPARγ is lost due to factors like aP2 and adiponectin, adipocyte differentiation is consistently perturbed. In addition, a decrease in fat mass, often leading to lipodystrophy. However, the impact on insulin sensitivity varies among different animal models, with some showing improved sensitivity while others experience worsened sensitivity, depending on the extent of PPARγ deficiency (53–56).

## 4 Future perspectives

Studies on GN as an ED are very few. It is a new vital area to study since GN intake on daily basis is almost inevitable. Worse still, a number of studies have endorsed the GN intake for diabetic patients due to its anti-diabetic properties. Chances are high that GN intake may exacerbate the medical condition instead of treating it. Clinical trials on obese people who take GN must be conducted so that there is a clear understanding of concentrations that exert the therapeutic and obesogenic effects.

## 5 Conclusion

GN is a phytoestrogen that is obesogenic regardless of its countless medicinal properties. Research shows that GN is an ED that exerts its effects by interacting with mainly PPARγ protein. The obesogenic effect is concentration and tissue dependent. It is well demonstrated that obesogen potentiates adipocyte differentiation, lipid accumulation, and transform BMSCs into adipocytes. All these findings show that GN can cause obesity by interacting with PPARγ.

## Author contributions

JX: Writing – original draft. RM: Writing – original draft. PD: Writing – original draft. OM: Writing – original draft. IS: Writing – original draft. YS: Writing – original draft. YH: Writing – review & editing. ML: Writing – review & editing. TH: Writing – review & editing. BH: Writing – review & editing. WZ: Writing – review & editing. ST: Writing – review & editing.
